# Identification of C3 and FN1 as potential biomarkers associated with progression and prognosis for clear cell renal cell carcinoma

**DOI:** 10.1186/s12885-021-08818-0

**Published:** 2021-10-23

**Authors:** Yang Dong, Wei-ming Ma, Wen Yang, Lin Hao, Shao-qi Zhang, Kun Fang, Chun-hui Hu, Qian-jin Zhang, Zhen-duo Shi, Wen-da Zhang, Tao Fan, Tian Xia, Cong-hui Han

**Affiliations:** 1 Department of Urology, Xuzhou Central Hospital, Xuzhou, China; 2 Medical College of Soochow University, Suzhou, China; 3 Department of Nephrology, The First Affiliated Hospital of Shandong Academy of Medical Sciences, Jinan, China; 4 Nanjing University of Traditional Chinese Medicine, Nanjing, China; 5 Department of Urology, The Third Affiliated Hospital of Soochow University, Changzhou, China; 6 Jiangsu Normal University, Xuzhou, China

**Keywords:** Clear cell renal cell carcinoma, Biomarker, Prognostic value, Complement component 3, Fibronectin 1

## Abstract

**Background:**

Clear cell renal cell carcinoma (ccRCC) is one of the most lethal urological malignancies, but the pathogenesis and prognosis of ccRCC remain obscure, which need to be better understand.

**Methods:**

Differentially expressed genes were identified and function enrichment analyses were performed using three publicly available ccRCC gene expression profiles downloaded from the Gene Expression Omnibus database. The protein-protein interaction and the competing endogenous RNA (ceRNA) networks were visualized by Cytoscape. Multivariate Cox analysis was used to predict an optimal risk mode, and the survival analysis was performed with the Kaplan-Meier curve and log-rank test. Protein expression data were downloaded from Clinical Proteomic Tumor Analysis Consortium database and Human Protein Atlas database, and the clinical information as well as the corresponding lncRNA and miRNA expression data were obtained via The Cancer Genome Atlas database. The co-expressed genes and potential function of candidate genes were explored using data exacted from the Cancer Cell Line Encyclopedia database.

**Results:**

Of the 1044 differentially expressed genes shared across the three datasets, 461 were upregulated, and 583 were downregulated, which significantly enriched in multiple immunoregulatory-related biological process and tumor-associated pathways, such as HIF-1, PI3K-AKT, P53 and Rap1 signaling pathways. In the most significant module, 36 hub genes were identified and were predominantly enriched in inflammatory response and immune and biotic stimulus pathways. Survival analysis and validation of the hub genes at the mRNA and protein expression levels suggested that these genes, particularly complement component 3 (C3) and fibronectin 1 (FN1), were primarily responsible for ccRCC tumorigenesis and progression. Increased expression of C3 or FN1 was also associated with advanced clinical stage, high pathological grade, and poor survival in patients with ccRCC. Univariate and multivariate Cox regression analysis qualified the expression levels of the two genes as candidate biomarkers for predicting poor survival. FN1 was potentially regulated by miR-429, miR-216b and miR-217, and constructed a bridge to C3 and C3AR1 in the ceRNA network, indicating a critical position of FN1.

**Conclusions:**

The biomarkers C3 and FN1 could provide theoretical support for the development of a novel prognostic tool to advance ccRCC diagnosis and targeted therapy.

**Supplementary Information:**

The online version contains supplementary material available at 10.1186/s12885-021-08818-0.

## Background

As one of the major causes of death from cancer worldwide, renal cell carcinoma (RCC) accounts for 3% of all human cancers, which leads to over 100,000 deaths per year and remains refractory to treatment [1, 2]. Clear cell RCC (ccRCC), responsible for ~ 75% of all RCC cases, results in more deaths annually than other histological subtypes, making it one of the most fatal malignancies in urology [3, 4]. Small-size renal tumors are usually asymptomatic and non-malignant in > 30% of all cases. However, due to the lack of early-stage diagnosis and the fact that imaging often misidentifies non-malignant tumors, patients often undergo potentially harmful, unnecessary, and sometimes invasive treatments, such as kidney removal, only to discover that the postoperative pathological assessments showed a benign tumor [5]. Excision is the best treatment choice for patients with small renal carcinoma and usually improves survival. However, for patients with advanced or metastatic ccRCC, prognosis remains unfavorable [6], and most patients eventually succumb to the disease due to limited therapeutic options and resistance to chemotherapy and radiotherapy [7–9]. Presently, the cellular and molecular mechanisms underlying ccRCC pathophysiology are still not well understood. Therefore, identification of effective biomarkers that could predict the occurrence of ccRCC and increase the accuracy of prognosis is particularly essential, and may help reduce excess therapy and clinical monitoring, thereby facilitating the development of reliable early diagnostic options and effective therapeutic strategies.

**Fig. 1:**
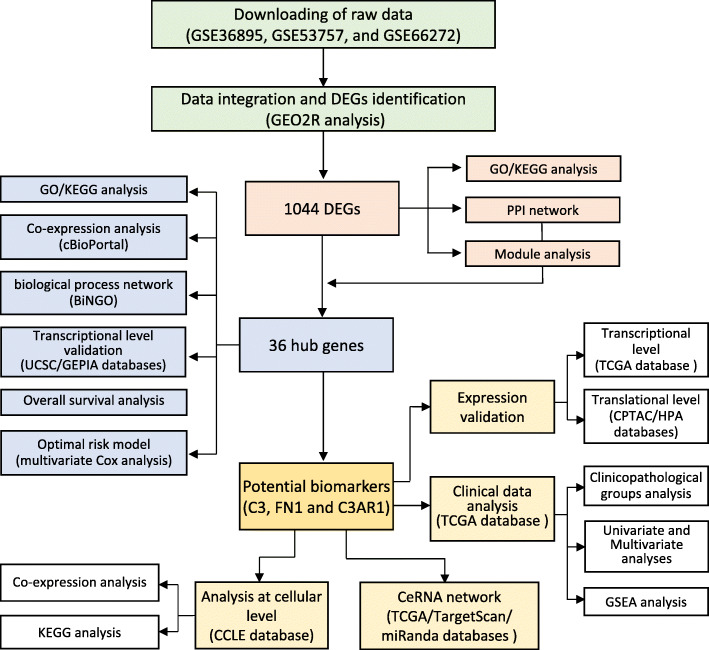
The workflow of data preparation, processing, analysis and validation in this study

Over the past few decades, microarrays based on high-throughput platforms have become powerful tools that have been widely applied for screening genetic alternations in carcinogenesis. Using gene expression profiling microarray analysis, researchers have identified nearly all differentially expressed genes (DEGs) in ccRCC, which are potentially useful for tumour diagnosis and prognosis. However, sample heterogeneity in independent studies or analyses in only a single cohort study has reduced the availability of reliable biomarkers in ccRCC. Therefore, we downloaded three original mRNA microarray datasets from the Gene Expression Omnibus (GEO) database and conducted a preliminary analysis to obtain DEGs between ccRCC and non-cancerous tissues. The biological effects of DEGs were subsequently assessed via Gene Ontology (GO) analysis, and the corresponding pathways were determined using the Kyoto Encyclopedia of Genes and Genomes (KEGG) database. Additionally, protein-protein interaction (PPI)-network analysis was performed as a novel approach to gain an in-depth understanding of the molecular mechanisms underlying the pathophysiology of ccRCC. Ultimately, 36 hub genes were identified, which we speculate are likely to include novel diagnostic biomarkers and therapeutic targets related to ccRCC. A schematic overview of the research workflow has been represented in Fig. 1.

## Methods

### Microarray data

We downloaded three gene expression datasets (GSE36895 [10], GSE53757 [11], and GSE66272 [12];S Affymetrix GPL570 platform, and Affymetrix Human Genome U133 Plus 2.0 Array) from the GEO database (http://www.ncbi.nlm.nih.gov/geo) [13]. The annotation information was referenced to convert the probes into the corresponding gene symbols. GSE36895 contained data from 29 ccRCC tissue samples and 23 tumor-adjacent tissue samples; GSE53757 from 72 ccRCC samples and 72 tumor-adjacent samples; and GSE66272 from 26 ccRCC samples and 26 tumor-adjacent samples. ccRCC patients in TCGA cohorts were also included in the study. The relevant mRNA expression, miRNA, and clinical data of ccRCC were downloaded from The Cancer Genome Atlas Kidney Renal Clear Cell Carcinoma (TCGA-KIRC) (https://cancergenome.nih.gov/; updated March 31, 2021). The mRNA and miRNA expression data that contained incomplete clinical information were excluded. Protein expression data were downloaded from Clinical Proteomic Tumor Analysis Consortium (CPTAC) databases (https://cptac-data-portal.georgetown.edu/; updated April 27, 2021). RNA expression (RNA-Seq) data of the hub genes in different renal cancer cell lines (*n* = 32) were obtained from the Cancer Cell Line Encyclopedia (CCLE) database (https://portals.broadinstitute.org/ccle/about; updated January 02, 2019) [14].

### Identification of DEGs

We used the GEO2R (http://www.ncbi.nlm.nih.gov/geo/geo2r) tool to screen for DEGs between ccRCC and tumor-adjacent samples in the GEO database. The adjusted P- (adj. P) values and the Benjamini–Hochberg procedure for determining the false discovery rate (FDR) were employed to balance the discovery of significant genes while limiting the number of false positives. Additionally, Limma package [15] in the R/Bioconductor software was used to perform the normalization and log^2^ conversion of the matrix of TCGA dataset. When a gene corresponded to more than one probe set, the average value of these probe sets was used. However, when the corresponding gene symbols were missing, the probe sets were removed. A log-fold change (logFC) value > 1 and an adj. *P*-value < 0.01 were usually considered to reflect a statistically significant difference in gene expression.

### GO and Kyoto encyclopedia of genes and genomes (KEGG) enrichment analyses

We used the DOSE [16] and clusterProfiler [17] packages of the statistical software R (Version 3.6.2) for mining information related to the biological effects of DEGs and for implementing KEGG pathway enrichment [18–20]. The ggplot2 and pROC packages were used for high-quality graph generation. Gene Set Enrichment Analysis (GSEA) 4.0.3 was used for GSEA analysis. The functional gene set file “c2.cp.kegg.v7.0.symbols.gmt” was used to summarize specific and well-defined signaling. The number of substitutions per analysis was set at 1000, and gene sets with *P* < 0.05 were recognized as significantly enriched.

### Protein-protein interaction (PPI) network construction and module analysis

We established the initial PPI network using the Search Tool for the Retrieval of Interacting Genes/Proteins (STRING; version 11.0; http://string-db.org) platform [21]. The minimum value for highest confidence was set to 0.7, and unconnected proteins were removed from the network. Molecular Complex Detection (MCODE) (version 1.4.2), an application plugin of Cytoscape (version 3.4.0), was applied to cluster a given network based on topology to identify densely connected regions [22]. Using the Cytoscape visualization software, we mapped the final PPI networks, and further identified the most significant module according to MCODE. The data were filtered based on the following criteria: MCODE score > 5, maximum depth = 100, node score cut-off =0.2, degree cut-off =2, and k-score = 2.

### Establishment of the hub gene networks

Genes with a degree of ≥10 were screened out as hub genes. Establishment of the hub gene networks and prediction of co-expressed genes were performed using the cBioPortal platform (http://www.cbioportal.org) [23, 24]. We used the Cytoscape visualization plugin Biological Networks Gene Ontology (BiNGO) tool (version 3.0.3) for visual representation of data [25]. Additionally, the hierarchical clustering of hub genes was performed using the University of California Santa Cruz (UCSC) Cancer Genomics Browser (http://genome-cancer.ucsc.edu) [26], and the mRNA expression levels in ccRCC samples were analyzed using the Gene Expression Profiling Interactive Analysis (GEPIA) platform [27]. Furthermore, overall survival (OS) and recurrence-free survival (RFS) analyses were performed based on the Kaplan-Meier analysis method. Translation-level candidate validation was conducted using the Human Protein Atlas (HPA) database (https://www.proteinatlas.org/), which provided information regarding the distribution of all human proteins in tissues and cells [28]. Moreover, relationships between expression patterns, tumor grades, and tumor stages were explored by reviewing the information in TCGA database.

### Analysis of potential regulators of the hub genes

We established a competing endogenous RNA (ceRNA) network. First, using the TargetScan (http://www.targetscan.org/) and miRanda (http://www.microrna.org/microrna/home.do) databases, we identified the potential miRNA of the target genes. Then, using the edgeR package in the R statistical environment, significant differentially expressed long non-coding RNAs (DElncRNAs) were identified in 539 ccRCC and 72 adjacent non-cancer renal tissues from TCGA database. | Log_2_FC | > 2.0 and FDR adjusted to *P* < 0.05 were set as the thresholds. The significant differentially expressed miRNAs (DEmiRNAs) were identified with the thresholds of |Log_2_ FC| > 1.0 and adj. *P*-value < 0.05 in 545 ccRCC and 71 adjacent non-cancer renal tissues from TCGA database. Using miRcode (http://www.mircode.org/), the DElncRNA-related DEmiRNA was predicted, while the DEmiRNA irrelevant to the hub genes were removed. Based on the interaction among lncRNA-miRNA-mRNA, the ceRNA network was established, which was visualized using Cytoscape.

### Statistical analysis

Discrete variables were expressed using a box plot to measure expression differences. The chi-square test was used to analyze the relationship between gene expression and clinical data. The Kaplan-Meier curve and log-rank test were used for plotting survival curves. Univariate Cox regression analysis was used to select relevant variables, and, subsequently, multivariate Cox regression analysis was used for the prognostic analysis of gene expression regarding the OS rate of ccRCC patients. Based on the expression level of each mRNA and the regression coefficient obtained through multivariate Cox regression analysis, a risk score was calculated as follows: Risk score = ExpmRNA1 × βmRNA1 + ExprnRNA2 × βmRNA2 + ⋯ + ExpmRNAn×βmRNAn (Exp represents the expression level of the mRNA and β represents the regression coefficient of the mRNA). An optimal risk model was developed based on the Akaike Information Criterion (AIC) [29]. According to the median value of risk scores, patients were divided into high-risk and low-risk groups. The area under the curve (AUC) of the receiver operating characteristic (ROC) curves were used to determine the predicted power of the prognostic gene signature. *P* < 0.05 was considered significant.

## Results

### Identification of DEGs in ccRCC patients

**Fig. 2:**
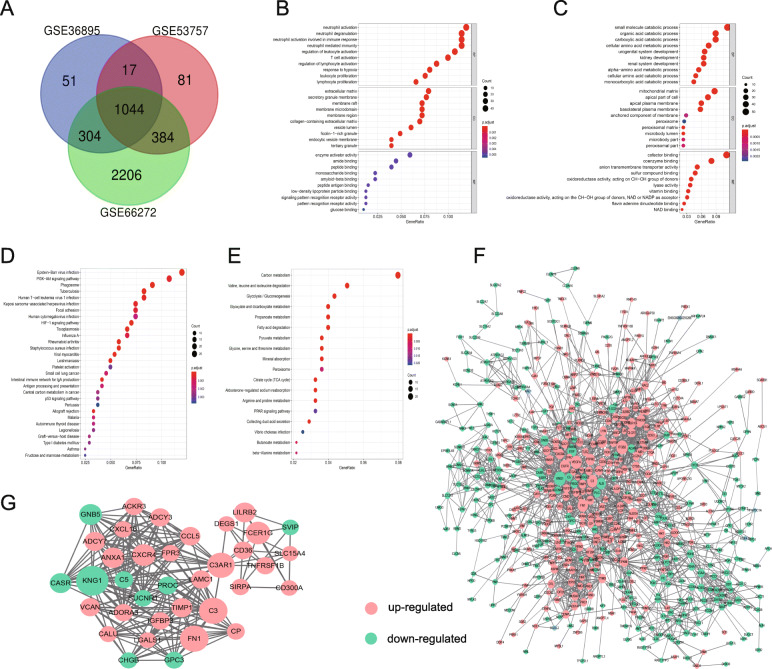
DEGs identification and enrichment analysis of hub genes. (**A**) DEGs with a fold change of > 2 and an adj. *P*-value < 0.01 were selected from the mRNA expression profiling sets GSE36895, GSE53757, and GSE66272. (**B**) Bubble plot of GO terms related to biological process (BP), cellular component (CC) and molecular function (MF) of upregulated DEGs and (**C**) downregulated DEGs. (**D**) Bubble plot of KEGG pathway analysis of upregulated DEGs and (**E**) downregulated DEGs. GO or pathway description was assigned to the y-axis and gene ratio was assigned to the x- axis axis as the proportion of differential genes in the whole gene set. The bubble size represents the gene count in a certain GO term or pathway. (**F**) The PPI network of DEGs was constructed using Cytoscape and DEGs with ≤3 nodes, and ≤ 3 edges were removed from the network. Each node size is indicated. (**G**) The most significant module obtained from the PPI network had 36 nodes and 218 edges. Upregulated genes are marked in light red, and downregulated genes are marked in light green

Analysis of the three gene expression datasets revealed 1416, 1526, and 3938 DEGs in GSE36895, GSE53757, and GSE66272, respectively. Comparing ccRCC tissues to tumor-adjacent tissues showed that 1044 genes shared across the three datasets, including 461 upregulated and 583 downregulated genes (Fig. 2A; Supplementary Table S1).

### GO classification and KEGG pathway enrichment analysis of DEGs

A total of 857 and 264 remarkably enriched GO terms (adj. *P-*value ≤0.05) were obtained for the upregulated and downregulated DEGs, respectively (Supplementary Table S2). The upregulated DEGs were mainly involved in the biological process of immune-related process, the regulation of immune cell activation and proliferation, and response to hypoxia and interferon-gamma. The downregulated genes were mainly enriched in the regulation of catabolic and metabolic process of multi-substances. Figure 2B and C represents the prior significantly enriched GO terms of upregulated and downregulated DEGs in each classification in the bubble graphs. The KEGG pathway analysis (Supplementary Table S2) indicated that the upregulated DEGs were significantly enriched in 60 terms, such as cell adhesion molecules, focal adhesion, ECM-receptor interaction, and HIF-1, PI3K-AKT, P53 and Rap1 signaling pathway; and the downregulated DEGs were remarkably enriched in 18 terms, such as carbon metabolism, glycolysis/gluconeogenesis, and PPAR signaling pathway, which were all related to the occurrence and development of ccRCC. Figure 2D and E represents the prior significantly enriched signaling pathways in the bubble graphs.

### Integration of PPI network and module analysis

**Fig. 3:**
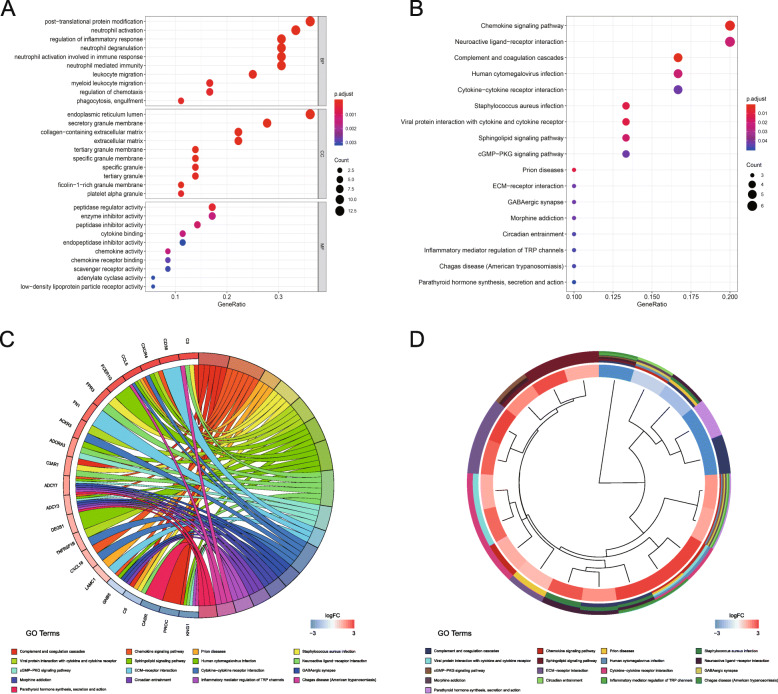
GO terms and KEGG pathway analysis results for hub genes. (**A**) Bubble plot of biological process (BP), cellular component (CC), and molecular function (MF) GO terms of hub genes. (**B**) Bubble plot for the KEGG pathway analysis of hub genes. GO term or pathway description was assigned to the y axis and gene ratio, as the proportion of differential genes in the whole gene set, was assigned to x axis. The bubble size represents the gene count of the corresponding GO term or pathway. (**C**) KEGG chord plot showing the relationship between enrichment pathways and genes, with chords linking each protein to the functions/processes it is related to. A gene is linked to a certain pathway by a colored chord, and blue-to-red coding next to the genes indicates logFC. (**D**) KEGG cluster of genes grouped by their functional categories. The inner ring shows the color-coded logFC, and the outer ring shows the assigned signaling pathways

The PPI network of DEGs (Fig. 2F) was constructed, and a hub module was mined, with 36 nodes and 218 edges (Fig. 2G, Supplementary Table S3). Following GO classification and KEGG pathway analyses of genes involved in this module, we obtained 369 and 17 remarkably (adj. *P-*value ≤0.05) enriched GO terms and pathways, respectively (Supplementary Table S4). Based on the results, thses genes were predominantly enriched in terms such as regulation of inflammatory and immune response and angiogenesis, response to biotic stimulus and wounding, protein modification, and phagocytosis. Besides, multiple tumor-related pathways were significantly enriched, including complement and coagulation cascades, chemokine and cGMP-PKG signaling pathway, ECM-receptor interaction, and cytokine-cytokine receptor interaction. The prior enriched GO terms and KEGG pathway were represented in bubble graphs (Fig. 3A and B). The chord plot represented all significantly enriched pathway, and the linked band indicated that a gene was in a certain term (Fig. 3C). The cluster plot displayed a circular dendrogram of the clustering of the expression profiles (Fig. 3D).

### Hub gene identification and analysis

**Fig. 4:**
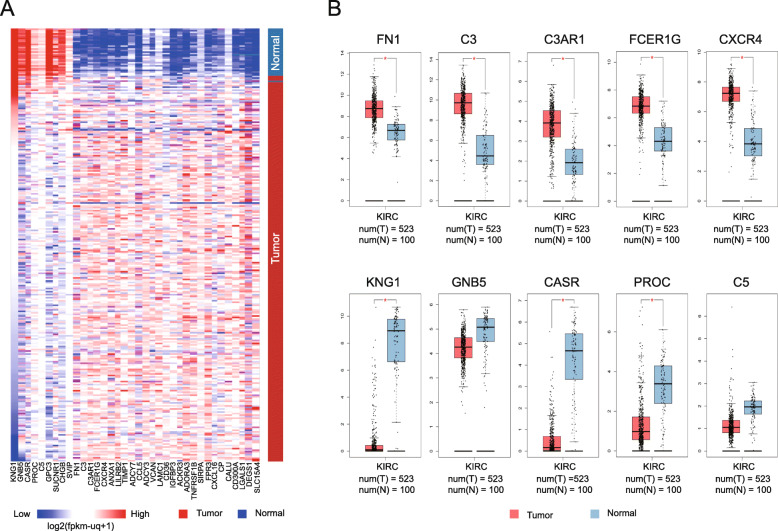
Transcriptional level validation of hub genes. (**A**) Hierarchical clustering of hub genes was performed using UCSC Cancer Genomics Browser. Upregulated genes are marked in red, and downregulated genes are marked in blue. (**B**) The mRNA levels of the top five upregulated and the top five downregulated hub genes obtained in ccRCC samples by reviewing the GEPIA database. *P < 0.01. The samples shown under the blue bar are non-cancerous samples, and the samples shown under the red bar are ccRCC samples

Genes mined in the hub module of the PPI network were retained as hub genes (Supplementary Table S3). According to the degree values, the top five upregulated genes were C3, FN1, C3AR1, FCER1G and CXCR4; the top five downregulated genes were KNG1, GNB5, CASR, PROC and C5. The co-expression network associated with hub genes was predicted based on cBioPortal and showed that some genes particularly C3, FN1and GNB5 acting as regulators were involved in the regulation of expression and state of downstream genes (Supplementary Fig. S1A); and the biological process network was also constructed suggesting an essential roles of hub genes in regulation of response to externa stimulus, wounding and inflammatory (Supplementary Fig. S1B). Hierarchical clustering using data extracted from UCSC database (Fig. 4A) revealed that the hub genes helped distinguish ccRCC tissues from non-cancerous tissues. The expression of the top five upregulated and downregulated hub genes was further confirmed in more ccRCC tissue samples in GEPIA online platform (Fig. 4B). Next, the correlation between the OS of the cancer patients and hub genes was analyzed and the results showed that ccRCC patients with altered expression of FN1, FCER1G, TIMP1, ADCY7, TNFRSF1B, CALU, LGALS1, and CASR showed worse overall survival (Supplementary Fig. S2A). Following multivariate Cox analysis of the hub genes was performed and an optimal risk model with nine genes was developed based on the AIC (Supplementary Table S5). Among them, LILRB2, TIMP1, ADCY7, ACKR3, and SIRPA showed positive coefficients, but C3AR1, CXCR4, IGFBP3, and CASR displayed negative coefficients. The median value of risk score for each sample was calculated to be 1.016 and used as the cut-off value. Based on the survival curve (Supplementary Fig. S2B), the five-year survival rate was 43.5% (95% confidence interval (CI), 36.3–52.2%) in the high-risk group (265 patients) and 75.3% (95% CI, 68.7–82.5%) in the low-risk group (265 patients). Simultaneously, an ROC curve was plotted (Supplementary Fig. S2C), and the AUC was 0.70, indicating that our model could well predict patient survival.

### C3, FN1, and C3AR1 expression at the transcriptional and translational levels

**Fig. 5:**
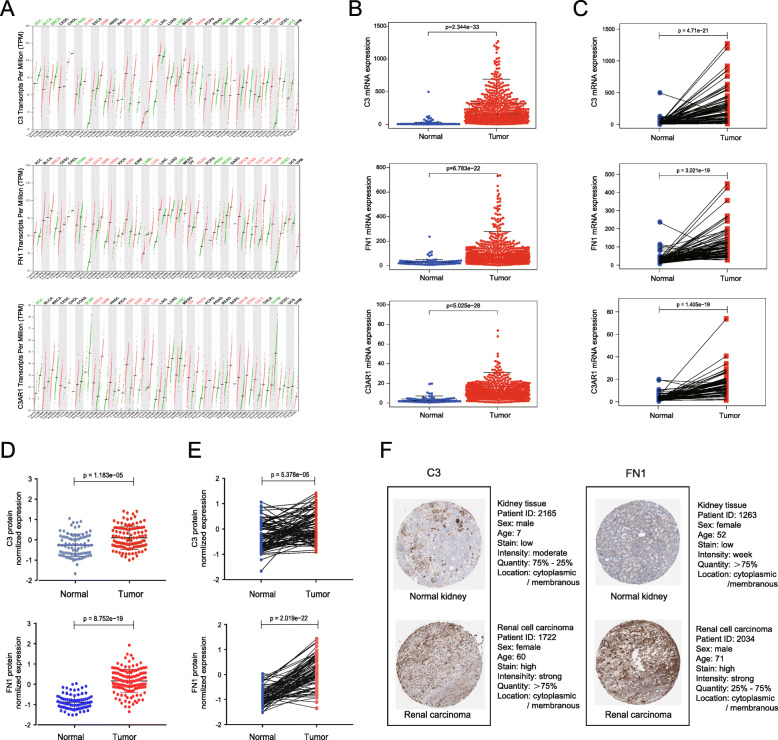
Validation of candidate gene expression levels at the transcriptional and translational levels. (**A**) C3, FN1, and C3AR1 mRNA expression in various normal human tissues and cancer tissues. (**B**) Comparison of C3, FN1, and C3AR1 mRNA expression levels in ccRCC tissues (*n =* 539) and normal renal tissues (*n =* 72). (**C**) Comparison of C3, FN1, and C3AR1 mRNA expression levels in 72 paired ccRCC tissues. (**D**) Comparison of C3 and FN1 protein expression levels in ccRCC tissues (*n =* 110) and normal renal tissues (*n* = 84). (**E**) Comparison of C3 and FN1 protein expression levels in 84 paired ccRCC tissues. (**F**) Representative IHC staining images of C3 and FN1 in normal kidney tissues and ccRCC tissues. *P < 0.05, **P < 0.01, ***P < 0.001

Analysis of the hub module of the PPI network revealed that C3, FN1, and C3AR1 were the most central regulators, indicating their possible vital roles in ccRCC development. The mRNA expression level of these genes in multiple healthy organ and tumor tissues was reviewed (Fig. 5A). C3, FN1, and C3AR1 were all highly expressed in ccRCC tissues (*n =* 539) than healthy renal tissues (*n =* 72) (*P <* 0.001, Fig. 5B). The same trend was observed in 72 paired ccRCC tissues to exclud the influence of individual differences (*P <* 0.001, Fig. 5C). Additionally, the protein levels of C3 and FN1, but not that of C3AR1, were significantly higher in ccRCC tissues than in normal kidney tissues (Fig. 5D, E), as suggested by CPTAC and HPA. The immunohistochemistry (IHC) staining results from HPA databases show that C3 and FN1 are typically located in the cytoplasm and membranes; however, C3AR1 was not detected in both normal renal and ccRCC tissues (Fig. 5F). These results suggested that C3 and FN1 were significantly upregulated both at the transcriptional and translational levels in ccRCC tissues, while C3AR1 was upregulated only at transcriptional level.

### Correlation of C3 and FN1 overexpression with tumor progression

**Fig. 6:**
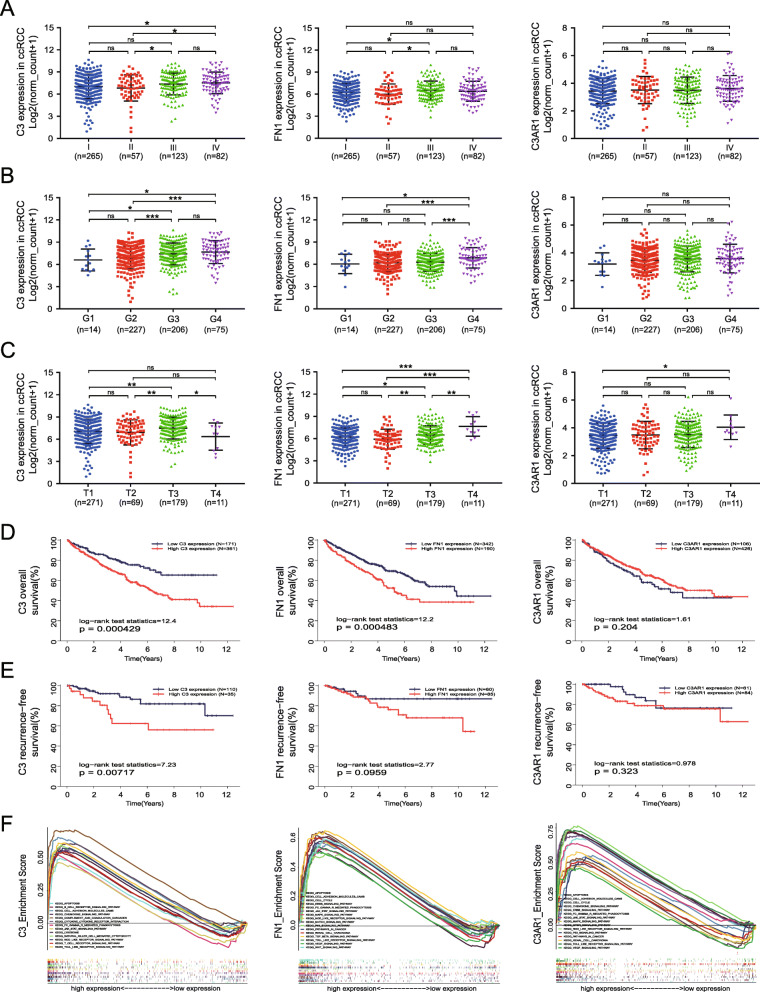
Comparison of candidate gene expression levels in different clinicopathological groups, survival analysis, and GESA analysis. Comparison of C3, FN1, and C3AR1 expression levels in groups of different (**A**) overall cancer stage, (**B**) histological grade (G), and (**C**) tumor size (T). Kaplan-Meier plots for (**D**) overall survival analysis and (**E**) recurrence-free survival analyses of the three genes in ccRCC based on the Kaplan-Meier plot in TCGA-KIRC dataset. (**F**) Representative signaling pathways significantly enriched in C3, FN1, and C3AR1 overexpression phenotypes determined by GSEA

Patients enrolled in this study (*n =* 532, TCGA database) were segmented into low and high target gene expression groups based on the optimal threshold of OS to investigate the clinical implication of C3, FN1 and C3AR1 expression. As shown in Table 1, C3 overexpression was significantly associated with advanced clinical stages; later stages of tumor, nodes, and metastases (TNM) classification; and high death rate (all *P <* 0.05). FN1 overexpression was significantly associated with males, advanced clinical stages, advanced tumor classification, and high death rate (all *P <* 0.05). However, no significant clinicopathological difference was obtained between the C3AR1 expression levels of the two target gene expression groups (Supplementary Table S6, all *P >* 0.05). Consistently, the difference in the C3 and FN1 expression levels between these different clinicopathological groups was further confirmed by analyzing mRNA expression data as continuous variable (Fig. 6A-C).

**Table 1:** Association between C3 and FN1 expression and demographic and clinicopathological parameters of patients with ccRCC in the TCGA profile

Parameter		C3 expression				FN1 expression			
		High (*n =* 361)	Low (*n* = 171)	χ^2^	*p*-value	High (*n* = 190)	Low (*n* = 342)	χ^2^	*p*-value
Age (mean ± SD)		60.96 ± 12.46	59.80 ± 11.43		0.304	59.28 ± 12.75	61.31 ± 11.74		0.065
Gender	Female	118	68	2.557	0.110	49	137	10.937	**0.001**
	Male	243	103			141	205		
Clinical stage	I/II	202	121	11.011	**0.001**	104	219	3.872	**0.049**
	III/IV	158	49			84	123		
	Unknown	1	1			2	0		
Pathologic T	T1,T2	215	126	10.064	**0.002**	108	233	6.761	**0.009**
	T3,T4	146	45			82	109		
	Unknown	0	0			0	0		
Pathologic N	N0	168	72		**0.046**	85	155	2.802	0.094
	N1	15	1			9	7		
	Nx + null	178	98			96	180		
Pathologic M	M0	284	138	3.894	**0.048**	152	270	0.577	0.448
	M1	62	17			32	47		
	Mx + null	15	16			6	25		
Recurrence status	No	71	50	0.533	0.466	33	88	0.036	0.850
	Yes	16	8			7	17		
	Null	274	113			150	237		
Living status	Living	34	252	14.467	**<0.001**	114	243	6.760	**0.009**
	Dead	15	85			76	99		

***Abbreviations*:** *ccRCC* clear cell Renal Cell Carcinoma, *TCGA* The Cancer Genome Atlas, *T* primary tumour, *N* regional lymph node, *Nx* regional lymph nodes are unknown, *M* distant metastasis, *Mx* distant metastasis is unknown; Significant associations are shown in boldface in the *p*-value column (*p*-value < 0.05)

### Prediction of OS by C3 and FN1 overexpression

The prognostic values of C3, FN1, and C3AR1 in ccRCC was first assessed by generating Kaplan-Meier curves based on the optimal thresholds. The ccRCC patients with high C3 or FN1 expression had poorer OS (all *P <* 0.05), and high C3 expression was also associated with significantly worse RFS. C3AR1 had no prognostic value in both OS and RFS (Fig. 6D, E). In the univariate model, age, clinical stage, TNM stage, and C3 and FN1 expression were significantly related to OS in ccRCC (all *P <* 0.05). Multivariate Cox regression analysis confirmed that C3 and FN1 overexpression could potentially serve as a predictor for poor survival (C3: hazard ratio (HR) =1.498 (1.036, 2.166), *P =* 0.032; FN1: HR =1.512 (1.110,2.058), *P =* 0.009) when taken together with advanced age, N classification, and metastasis (Table 2). Conversely, C3AR1 expression was not significantly related to OS, and its overexpression was not an independent indicator of unfavorable OS (Supplementary Table S7). Gene expression enrichment analysis was performed between datasets with low and high candidate gene expression; multiple signaling pathway related to cancer development were all enriched in samples with high C3, or FN1, or C3AR1 expression phenotype, such as JAK/STAT signaling, chemokine signalling, NOD like receptor signaling, FC gamma R-mediated phagocytosis, and T and B cell receptor signaling pathways (Fig. 6F, Supplementary Table S8).

**Table 2:** Univariate and multivariate analyses of the overall survival in patients with ccRCC

Parameters	Univariate analysis				C3 - Multivariate analysis				FN1 - Multivariate analysis			
	*P-*value	HR	95% CI (lower/upper)		*P-*value	HR	95% CI (lower/upper)		*P-*value	HR	95% CI (lower/upper)	
Age	**<0.001**	1.775	1.307	2.410	**0.001**	1.714	1.259	2.333	**<0.001**	1.759	1.292	2.394
> 60 vs ≤60												
Female vs. male	0.711	1.060	0.779	1.442								
Clinical stage	**<0.001**	3.839	2.800	5.264	**0.013**	2.377	1.200	4.711	**0.006**	2.591	1.306	5.144
III/IV vs I/II												
Pathologic T	**<0.001**	3.156	2.333	4.269	0.891	0.959	0.529	1.739	0.701	0.889	0.488	1.619
T3/T4 vs T1/T2												
Pathologic N	**<0.001**	3.857	2.089	7.124	**0.027**	2.049	1.085	3.871	**0.036**	1.980	1.044	3.755
N1 vs N0												
Pathologic M	**<0.001**	4.387	3.222	5.973	**<0.001**	2.454	1.692	3.558	**<0.001**	2.372	1.635	3.442
M1 vs M0												
C3 expression	**0.001**	1.900	1.321	2.731	**0.032**	1.498	1.036	2.166				
High vs low												
FN1 expression	**<0.001**	1.695	1.256	2.288					**0.009**	1.512	1.110	2.058
High vs low												

***Abbreviations*:** *ccRCC* clear cell renal cell carcinoma, *HR* hazard ratio, *CI* confidence interval; Significant associations are shown in bold face in the *P*-value column (*P* < 0.05)

### Construction of ceRNA network

**Fig. 7:**
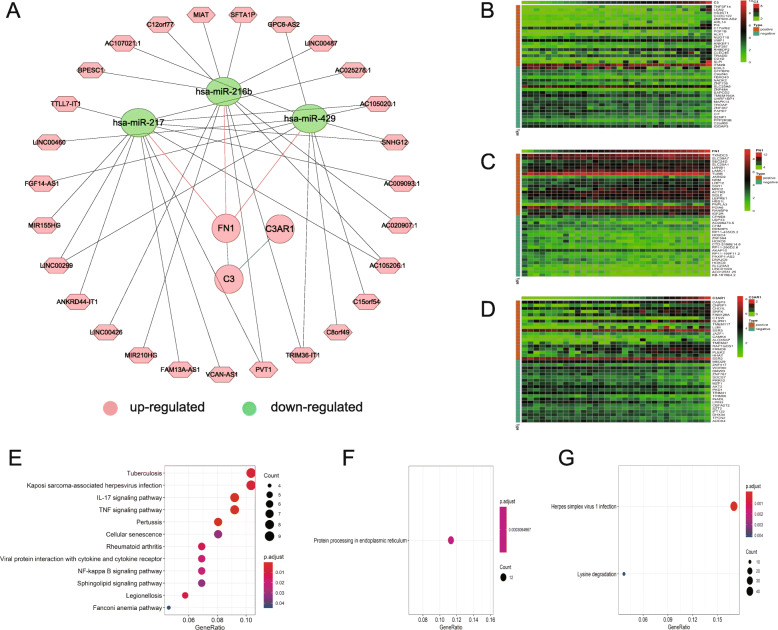
Construction of ceRNA Network and analyses of FN1 and C3 in renal cancer at the cellular level. (**A**) ceRNA network of C3, FN1, and C3AR1, (**B**) Heatmap of the top 20 genes co-expressed with C3, (**C**) FN1, and (**D**) C3AR1 in both upregulated and downregulated groups. The data used were extracted from the 32 renal cancer cell samples in the CCLE database. Orange indicates a positive correlation and green indicates a negative correlation between the candidate gene and the co-expressed genes. (**E**) Bubble plot of KEGG pathway analysis of the genes co-expressed with C3, (**F**) FN1, and (**G**) C3AR1. The bubble size represents the gene count for a certain GO term or pathway

We identified significant DElncRNAs and DEmiRNAs between ccRCC and adjacent non-cancer renal tissues in TCGA database, and obtained 1483 DElncRNAs (1044 upregulated and 439 downregulated) and 173 DEmiRNAs (106 upregulated and 67 downregulated) (Supplementary Fig. S3). Furthermore, miRNAs related to C3 (*n =* 1), C3AR1 (*n =* 3) and FN1 (*n =* 38) were predicted by intersecting the results obtained in TargetScan and micRnada databases (Supplementary Table S9). After identifying the miRNAs that had different regulatory effects on the candidate genes and lncRNAs, a ceRNA network was constructed, including 27 lncRNA nodes, 3 miRNA nodes, and 3 nodes of FN1, C3, and C3AR1 (Fig. 7A). As analyzed in ceRNA network, FN1 indirectly regulated the expression of multiple lncRNAs through binding with miR-429, miR-216b and miR-217, and constructed a bridge to C3 and C3AR1 which have indirect relationship with downregulated miRNAs, indicating a critical position of FN1.

### Analyses of FN1 and C3 in renal Cancer at the cellular level

*C3, FN1*, and *C3AR1* are overexpressed in multiple tumor cell lines (Supplementary Fig. S4). Using the co-expression tool on expression data extracted from renal cancer cell samples of CCLE, we obtained lists of genes that were co-expressed with FN1, C3, and C3AR1 and harbored a correlation coefficient > 0.5 or < − 0.5 and *P-*value < 0.01 (Supplementary Table S10). The expression data of top 20 related genes in both upregulated and downregulated groups were depicted in heatmaps (Fig. 7B-D). KEGG pathway analyses were conducted (Fig. 7E-G), and it is noteworthy that the genes co-expressed with C3 were remarkably enriched in IL-17 signaling, TNF signaing, and NF-κB signaling pathways; the genes co-expressed with FN1 were mainly enriched in protein processing in endoplasmic reticulum, which were all related to the occurrence and development of cancer.

## Discussion

RCC is estimated to rank fourteenth among the most common malignancies worldwide, with at least 400,000 new cases confirmed in 2018 [30]. Despite rapid advances in surgical therapy and clinical drug treatment, the overall mortality and five-year survival remain disappointing [6]. The mortality rate of RCC is predominantly due to difficulties in early diagnosis and a lack of efficient therapeutic methods for patients with advanced or metastatic status [7, 8]. Therefore, finding specific biomarkers for effective RCC diagnosis and treatment has become an urgent need.

Herein, three original mRNA microarray datasets were analyzed, which enabled the identification of 1044 DEGs between ccRCC and non-cancerous tissues. Classification of the biological functions and signaling pathways using GO and KEGG enrichment analyses of the DEGs revealed that these genes were mainly involved in immunological processes, including the regulation of various immune cells’ activation and proliferation and the hypoxia response pathway. These DEGs were also associated with ccRCC occurrence and development, such as cell adhesion molecules, focal adhesion, ECM-receptor interaction, and HIF-1, PI3K-AKT, P53, and Rap1 signaling pathways. Cell adhesion molecules can mediate cellular adhesion to control cell motility and transduce intracellular signaling [31], and focal adhesion also can control cell morphology, adhesion, and migration by connecting the ECM and intercellular F-actin [32], both of which are accordingly important for cancer invasion and metastasis, and are critical determinants in cancer cell resistance to therapy [33]. There is evidence that a positive feedback loop between the PI3K-AKT and HIF-1 pathways is involved in ccRCC tumorigenesis [9]. HIF-1 as a critical driver in ccRCC pathogenesis is commonly upregulated following von Hippel Lindau (VHL) mutations in RCC [34, 35]. Overexpression of HIF-1 promotes the expression of multiple growth factors, including VEGF, PDGF, and EGF. These factors in turn may activate the PI3K/AKT pathway via receptor tyrosine kinases (RTKs), leading to subsequent activation of mammalian target of rapamycin complex 1 (mTORC1) mTORC2, which then further promote HIF expression to contribute to ccRCC [9, 34]. P53 has been extensively studied in various cancers, which can function as a tumor suppressor, activating the expression of the pro-apoptotic protein, Bax, and suppressing the anti-apoptotic protein, Bcl-2 [36]. Increased P53 expression is also closely associated with metastasis and worse prognosis for patients with ccRCC [37]. However, the role of Rap1 has been reported to differ in malignancy according to the cancer types. A recent study on hepatocellular carcinoma progression revealed that Rap1 signaling pathway serves an critical role in the EMT phenotype, growth and apoptosis mediation of cancer cells [38]. RAP1 was also documented highly expressed in ccRCC and positively related to grades [39], and Rap1 signaling pathway was involved in enhancing the sensitivity of ccRCC to sunitinib treatment [40].

Based on the PPI network of DEGs, 36 hub genes were identified, which were predominantly related to the regulation of response to inflammatory, biotic stimuli, immune and multiple immune- and tumor-related pathways, including complement and coagulation cascades, chemokine and cytokine-cytokine receptor interaction, and cGMP-PKG signaling pathway. Evidences indicate that cGMP-PKG signaling pathway serves an essential role in cell proliferation and apoptosis in both colon cancer [41] and renal carcinoma cells [42]. In most pathophysiological situations, response to inflammation and various biotic stimuli are commonly associated with infiltration and activation of immune cells, which involves stimulation of protein cascades including the complement and coagulation systems, a burst of cytokine and chemokine production and cytokine-cytokine receptor interaction [43]. The immune system is involved in various tumor-related biological processes, and immunotherapy is one of the most potent modalities for treating renal tumors. The complement system, being a part of the immune system, also affects carcinoma development. C3, C3AR1, and C5 participate in the genesis and development of multiple tumor types [44, 45], while their effects on ccRCC remain obscure. The diversity of complement protein regulation creates a microenvironment favorable for cancer progression, which corroborates our results. Here, unlike C3AR1, C3 mRNA overexpression was significantly associated with advanced clinical stage, later stages of TNM classification, and poor OS and RFS rates in ccRCC patients, indicating a vital role of C3 in ccRCC carcinogenesis and progression. Therefore, C3 can potentially serve as a predictor for poor prognosis, while C3AR1 cannot.

FN1, a gene associated with adhesion and extracellular matrix remodeling, suppresses apoptosis, promotes epithelial cell migration, and drives tumor development in various cancers [46]. Higher *FN1* mRNA expression has been observed in renal tumor tissues, thus demonstrating the potential prognostic value of FN1 in renal cancer patients [47]. Additionally, cytoplasmic FN1 is significantly associated with advanced tumor stage and OS rate [48]. Moreover, in most cases, early tumors have high FN1 protein expression that does not change with stage progression [49]. Herein, increased FN1 correlated with an advanced clinical stage, a high pathological grade, and high death rate in ccRCC patients, but no significant correlation was found between FN1 expression trend and patient age, lymph node metastasis, distant metastasis, and recurrence status. Further univariate and multivariate analyses with subsequent Cox regression analysis qualified FN1 as a viable candidate biomarker for predicting poor survival. In our ceRNA network analysis, FN1 was found to directly interact with C3 and be regulated by miR-127, miR-216b, and miR-429, which have been reported to be downregulated in various tumors and act as tumor suppressors in RCC [50–52]. Their effects on tumor cells have been confirmed experimentally with respect to the WNT, HIF, PI3K/AKT, MAPK, and TGF-β pathways [50–54]. Herein, all these pathways, as well as apoptosis, cell cycle, and immune cell receptor signaling pathways were detected to be significantly enriched in the FN1 overexpression phenotype, which provided a theoretical basis for the ceRNA regulatory network involved in driving ccRCC progression through FN1.

FCER1G, a crucial molecule involved in allergic reactions, regulates cell apoptosis [55] and is positively correlated with the prognosis of patients with advanced ccRCC [56]. Chemokines and their receptors are important factors in the process of immune regulation. CXCR4 overexpression in renal cancer cells increases invasiveness, whereas CXCR4 silencing inhibits RCC cell growth and metastasis [57]. GNB5, an important mediator of intercellular and intermolecular information transfer, influences various physiological and pathological processes. Suppressing GNB5 increases cetuximab sensitivity in colorectal cancer cells and has been suggested as a combination therapy with cetuximab in cancer treatment [58]. Altered blood coagulation function is associated with multiple pathophysiological processes in tumors. As a natural anticoagulant, PROC limits extravasation, promotes cancer cell migration, and reduces the overall microenvironment’s clottability [59]. Additionally, CASR overexpression promotes RCC cell proliferation and migration and can be regarded as a novel prognostic biomarker for predicting RCC bone metastasis [60]. In our biological network module analysis, KNG1 showed the highest number of links with other nodes among downregulated genes. KNG1 in ccRCC patients is decreased at both the transcriptional and translational levels [5], but its effect on ccRCC has not been studied in depth. Furthermore, KNG1 has been suggested as a potential biomarker for early colorectal cancer diagnosis [61]. Additionally, this gene is involved in proinflammatory roles [62], angiogenesis [63], and apoptosis [64]. These findings provide evidence for the crucial role of KNG1 in tumor pathology.

The survival analysis for the hub genes showed that the alteration in the expression of *FN1, FCER1G, TIMP1, ADCY7, TNFRSF1B, CALU, LGALS1*, and *CASR* was involved in worsening of OS, thereby indicating vital roles of these genes in ccRCC carcinogenesis, progression, or invasion. TIMP1 is a natural inhibitor of metastasis-associated matrix metallopeptidases; its expression is correlated with poor survival ccRCC [65] and RCC invasion and migration in vitro [66]. ADCY7 plays a role in the CXCR4 pathway, and its deficiency has been found in patients with renal cancer-related muscle atrophy [67]. The TNF receptor, TNFRSF1B, has been associated with the progression of esophageal carcinoma, non-small cell lung cancer, and breast cancer [68]. CALU has the potential to serve as an effective target for inhibiting melanoma growth, invasion, migration, and metastasis [69]. LGALS1 is a potentially useful biomarker for renal cancer and is implicated in tumor progression and poor outcomes through the HIF/mTOR signaling axis in RCC patients [70].

Notably, our cBioPortal online analysis did not reveal any significant correlation between genetic alteration in C3 and shorter survival time. We speculate that this may be attributed to the fact that the cBioPortal survival analysis is based on the relationship between gene mutation and prognosis, while gene overexpression is usually caused by mutation or amplification. Therefore, C3 overexpression in ccRCC may be due to gene amplification rather than mutation, and further studies are needed to confirm this hypothesis. Additionally, multivariate Cox analysis of all hub genes was performed, and an optimal model was constructed, which included nine genes: *LILRB2, TIMP1, ADCY7, ACKR3, SIRPA, C3AR1, CXCR4, IGFBP3,* and *CASR*. The AUC of the ROC curve was 0.70, thus indicating that this model, which is based on calculating the risk scores of these nine genes, could predict patient survival well and be used as a prognostic tool.

There were several limitations to this study. First, there was lack of experiment for validation of our results. Second, the samples size integrated form the three datasets with a total of 127 ccRCC tissues and 121 tumor-adjacent samples remain still relatively limited, and the genetic data were mainly from patients in European and American regions (from the United States: GSE36895 and GSE53757; from Germany: GSE66272), lacking other ethnic and geographical diversity, all of which are likely to produce effects on our analysis of gene expression in ccRCC. Additionally, characteristics, as gender, age, pathological typing were not accounted for in this study, which may result in some potential biological information missed here.

## Conclusions

In summary, this study was designed to identify individual candidate biomarkers for early ccRCC diagnosis and prognostics. Through integrated bioinformatics analyses, 36 hub genes were selected for further study. Our findings provide novel insights into the molecular mechanisms through which oncogenic alterations drive ccRCC carcinogenesis and progression. The candidate biomarkers, particularly C3 and FN1, as well as the signalling pathways identified, could serve as therapeutic targets for ccRCC. The findings of this study provide preliminary theoretical support for developing novel prognostic methods for advancing ccRCC diagnosis and personalised therapy; however, further studies are needed in this direction.

## Supplementary Information

**Additional file 1.**

**Additional file 2.**

## Data Availability

The datasets used and/or analysed during the current study are available from the corresponding author on reasonable request. Publicly available datasets were analyzed in this study. This data can be found here: https://www.ncbi.nlm.nih.gov/geo/query/acc.cgi?acc=GSE36895 (accessed on 31 July 2019); https://www.ncbi.nlm.nih.gov/geo/query/acc.cgi?acc=GSE53757 (accessed on 25 March 2019); https://www.ncbi.nlm.nih.gov/geo/query/acc.cgi?acc=GSE66272 (accessed on 24 July 2019); https://cancergenome.nih.gov/ (accessed on 31 March 2021); https://cptac-data-portal.georgetown.edu/ (accessed on 27 April 2021); https://portals.broadinstitute.org/ccle/about (accessed on 02 January 2019).

## Abbreviations

BiNGO: Biological Networks Gene Ontology
BP: Biological processes
CC: Cell component
CCLE: Cancer Cell Line Encyclopedia
ceRNA: Competitive endogenous RNA
CI: Confidence interval
DAVID: Database for Annotation, Visualization and Integrated Discovery
DEGs: Differentially expressed genes
DElncRNAs: Differentially expressed lncRNAs
DEmRNAs: Differentially expressed microRNAs
FC: Fold change
FDR: False-discovery rate
GEO: Gene Expression Omnibus
GEPIA: Gene Expression Profiling Interactive Analysis
GO: Gene Ontology
GSEA: Gene set enrichment analysis
HPA: Human Protein Atlas
HR: Hazard ratio
KEGG: Kyoto Encyclopedia of Genes and Genomes
MCODE: Molecular Complex Detection
MF: Molecular function
MIBC: Muscle-invasive bladder cancer
NES: Normalised enrichment score
NMIBC: Non–muscle-invasive bladder cancer
OS: Overall survival
PPI: Protein–protein interactionRCC
RCC: Renal cell carcinoma
ROC: Receiver operating-characteristic
STRING: Search Tool for the Retrieval of Interacting Genes
TCGA: The Cancer Genome Atlas
