# Supplementary material for: Identification of C3 and FN1 as potential biomarkers associated with progression and prognosis for clear cell renal cell carcinoma
Source: BMC Cancer. 2021 Oct 23;21:1135. doi: 10.1186/s12885-021-08818-0 (PMC8539775; doi:10.1186/s12885-021-08818-0)
Supplement: Supplementary file 1 — Additional file 1. [file 12885_2021_8818_MOESM1_ESM.pdf]

Identification of C3 and FN1 as potential biomarkers associated with progression and prognosis for clear cell renal cell carcinoma

Yang Dong, Wei-ming Ma, Wen Yang, Lin Hao, Shao-qi Zhang, Kun Fang, Chun-hui Hu, Qian-jin Zhang, Zhen-duo Shi, Wen-da Zhang, Tao Fan, Tian Xia, Cong-hui Han。

Additional File 1. Supplementary Figures:

Supplementary Figure S1. Interaction network and biological process analysis of hub genes.

Supplementary Figure S2. Overall survival analyses for the hub genes.

Supplementary Figure S3. DElncRNAs and DEmiRNAs in patients with ccRCC.

Supplementary Figure S4. ***C3, FN1*** **and** ***C3AR1*** **expression in various cancer cell lines.**

Supplementary Figure S1.

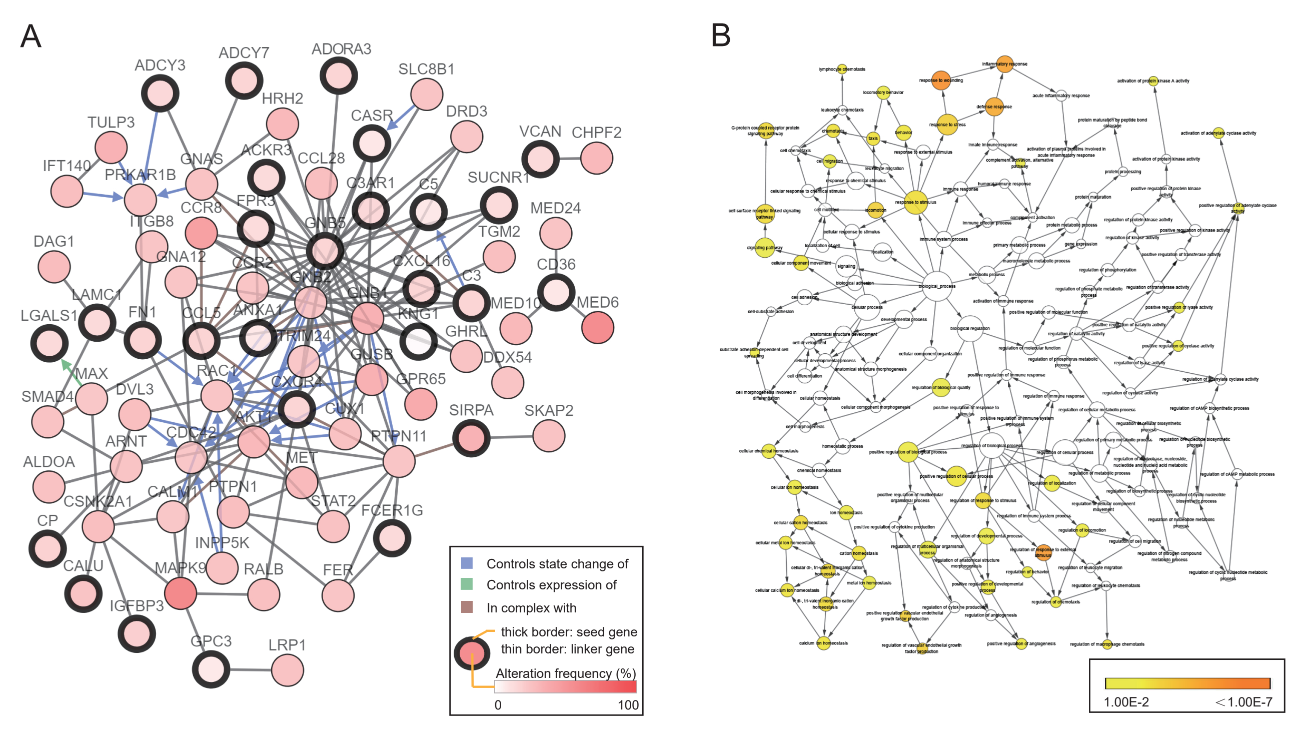

Supplementary Figure S1. Interaction network and biological process analysis of hub genes. (A) Hub genes and co-expressed genes were analysed using cBioPortal. Nodes with a bold black outline represent hub genes. Nodes with a thin black outline represent co-expressed genes. (B) Biological process analysis for hub genes was performed using BiNGO. The colour depth of each node indicates the corrected P-value for each ontological category. The node size indicates the number of genes involved in the ontologies. P < 0.01 was considered to reflect a statistically significant difference.

Supplementary Figure S2.

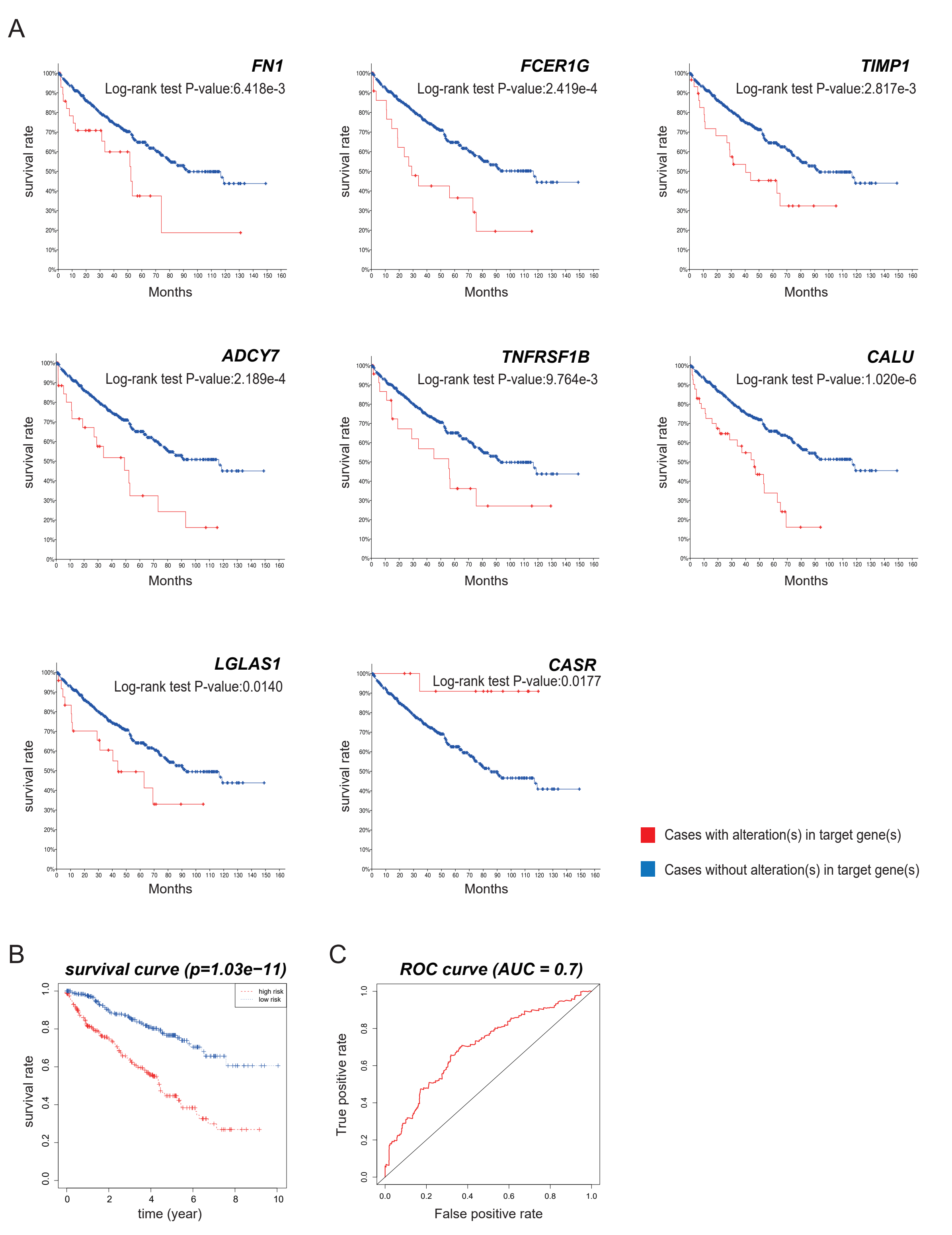

Supplementary Figure S2. Overall survival analyses for the hub genes. (A) Overall survival analyses for the hub genes was performed using the cBioPortal online platform and the significant ones were listed. (B) Survival curve Figure. The patients were divided into high-risk group (>1.016) and low-risk group (<1.016) according to the risk score. The difference in the survival rate between the high-risk and low-risk groups was significant (P<0.001). (C) ROC curve Figure. In the ROC curve analysis, it was obtained that AUC was 0.7. The larger the AUC value is, the more likely the current classification algo¬rithm is to place the positive sample in front of the negative sample.

Supplementary Figure S3.

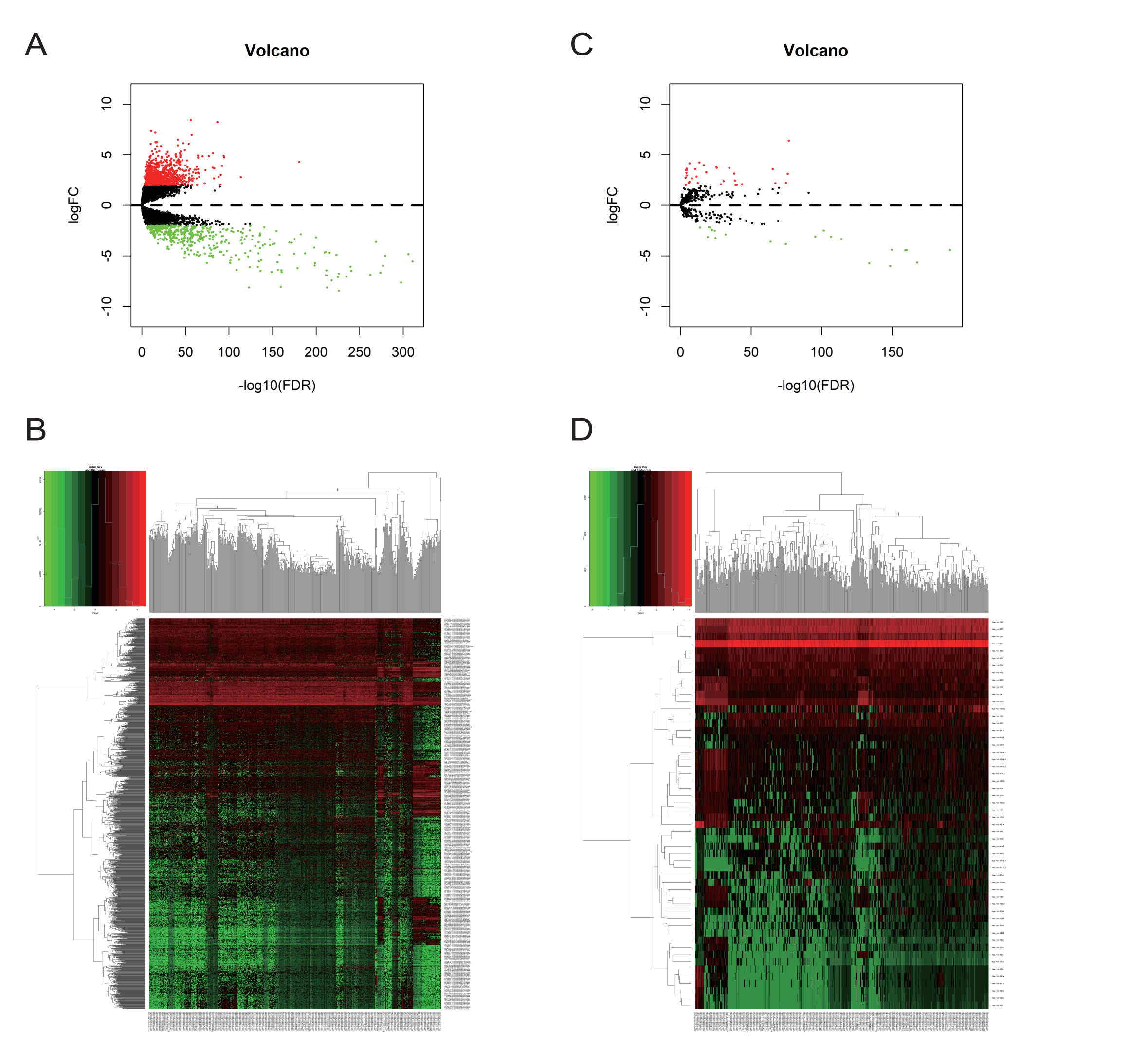

Supplementary Figure S3. DElncRNAs and DEmiRNAs in patients with ccRCC. (A) Volcano plot and (B) heatmap of DElncRNAs in ccRCC and adjacent non-carcinoma renal tissues. The DElncRNAs were identified with the thresholds of |Log2 FC| > 2.0 and adj.P-value < 0.05. (C) Volcano plot and (D) heatmap of DEmiRNAs in ccRCC and adjacent non-carcinoma renal tissues. The DEmiRNAs were identified with the thresholds of |Log2 FC| > 1.0 and adj.P-value < 0.05.

Supplementary Figure S4.

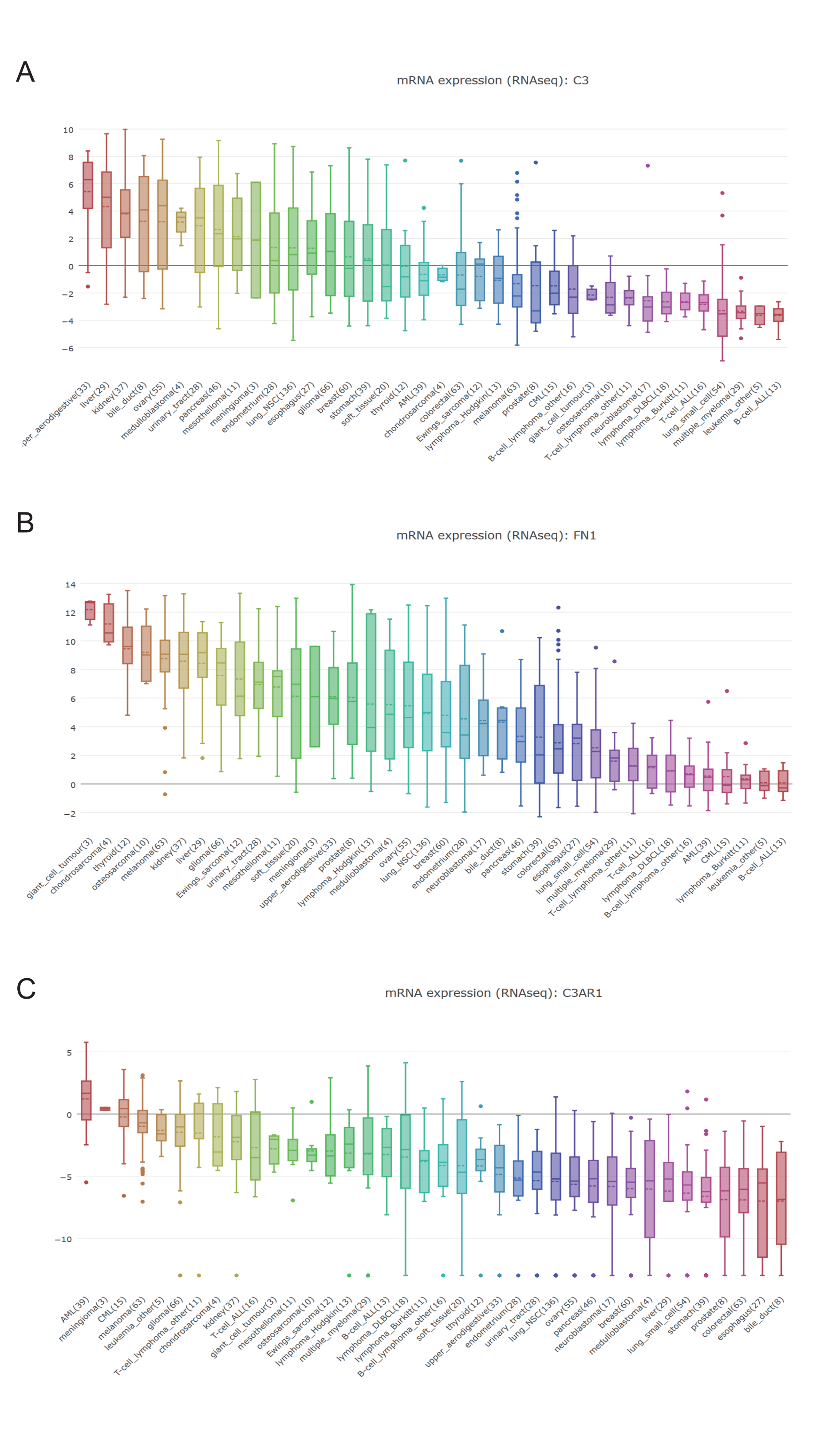

Supplementary Figure S4. ***C3, FN1*** **and** ***C3AR1*** **expression in various cancer cell lines.** (A) *C3* (B) *FN1* and (C) *C3AR1* mRNA-expression in various cancer cell lines obtained from Cancer Cell Line Encyclopedia database. The abscissa is the tumor type and sample size, and the ordinate is the expression of target genes.
